# A ‘giant’ paraganglioma in the testis

**DOI:** 10.1530/EDM-14-0055

**Published:** 2014-10-01

**Authors:** Marinos C Makris, Konstantinos C Koumarelas, Apostolos S Mitrousias, Giannos G Psathas, Athanasios Mantzioros, Stratigoula P Sakellariou, Panagiota Ntailiani, Evripides Yettimis

**Affiliations:** 1 First Surgical Department of General Hospital of Athens ‘Georgios Gennimatas’, Athens, Greece; 2 Alpha Institute of Biomedical Sciences (AIBS), Marousi, Athens, Greece; 3 Department of Pathology, Medical School, University of Athens, Athens, Greece

## Abstract

Until now, less than ten cases of extra-adrenal chromaffin cell tumors have been reported to be localized to the spermatic cord area. All published studies report benign tumors with a diameter <2–3 cm and no invasion of the testis. In this article, we present one case of a giant malignant paraganglioma in the testis of a patient who had initially been operated for a giant mass in the scrotum. The mass developed in approximately 4 months. This is the first study reporting the following findings: i) paraganglioma was found exclusively in the testis, invading the testicle and not the spermatic cord, ii) it was malignant with lung metastasis, and iii) its size was 17.5 cm×10 cm×9.5 cm. We present the first – giant – malignant paraganglioma. Moreover, it is the first case report of a paraganglioma in the testis.

**Learning points:**

- This is the first study reporting the following findings:
- Paraganglioma found exclusively in the testis, invading the testicle and not the spermatic cord.
- It is malignant with lung metastasis.
- It is of the size 17.5 cm×10 cm×9.5 cm.

## Background

The detection of paragangliomas has been reported to be extremely rare in the literature. Initially, these masses of chromaffin cells originating from the neural crest had been introduced as extra-adrenal pheochromocytomas, due to their catecholamine secretory capability; however, paraganglioma has become the commonly accepted term in the past decade. The most common sites of these tumors are the abdomen, the retroperitoneum along the para-aortic axis, the thorax, the cervical area, and the head. The testis and the spermatic cord are very rare locations. The first report of this mass was documented in 1971 by Eusebi & Massarelli (1). Since then, only seven more cases are available in the medical databases (2). Herein, we report the detection of this unknown tumor in the testis of a 66-year-old man who had been operated for exploration of the right hemiscrotum.

## Case presentation

The patient, a 66-year-old man, was admitted with a 4-month history of a gradual increase, in size of a, non-tender, mobile, big mass of the right hemiscrotal sac, following heavy weight lifting. The mass had been causing mobility problems to the patient due to its great size. There were no history of urinary symptoms or trauma of the scrotum.

## Investigation

The first impression following the initial clinical examination was that of a non-complicated giant inguinoscrotal hernia. The left testicle was normal. There were no palpable inguinal lymph nodes. The routine hematological and biochemical markers were normal. He had a history of high blood pressure, psoriasis, and hepatitis B and C with two previous operations (umbilical hernia and left inguinal hernia). His family history was unremarkable.

## Treatment

The patient was operated for exploration of his right inguinal canal and the scrotal sac. There were no signs of hernia and the identification of the right testicle was not feasible, due to its encapsulation in a slid mass that was arising from the cord. The contents of the spermatic cord were ligated and cut. The lump was excised en block with very little blood loss and no significant hemodynamic changes under general anesthesia. There were no pre- or post-operative complications from surgical and anesthetic points of view. The patient was discharged 3 days after the operation. During his hospitalization, there were no disturbances in his blood pressure.

## Outcomes and follow-up

### Histology

**Figure 1:**
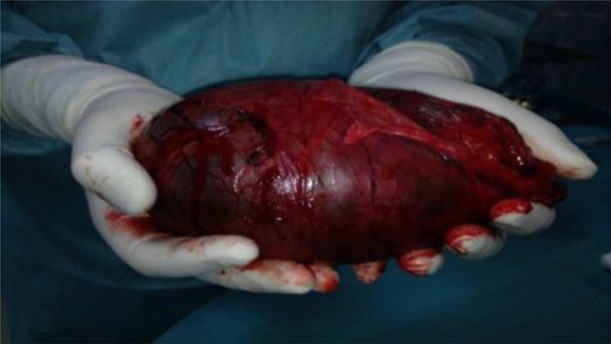
Macroscopic appearance of the excised tumor.

The histological examination following the excision showed the presence of a neuroendocrine tumor of size 17.5 cm×10 cm×9.5 cm (Fig. 1). The tumor was excised along with the spermatic cord (length 10 cm), whereas the histologically recognized right testicle was swollen to a size of 9 cm×5.8 cm×5.9 cm and invaded by the neoplastic tissue. The swollen testicle enclosed an abundance of fluid.

**Figure 2:**
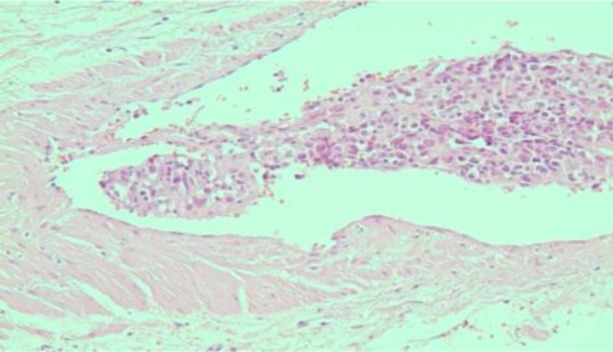
Slide of vascular neoplastic emboli.

**Figure 3:**
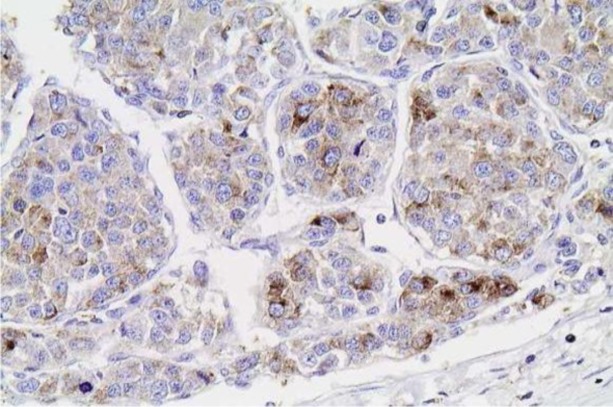
Cells positive for synaptophysin.

The tumor shows a nested architectural growth pattern with expanded vascular spaces between the neoplastic nests, resulting in macrotrabecular morphology. Focal and confluent necroses are encountered. Neoplastic cells are medium to large in size, partially polygonal with plenty of eosinophilic cytoplasm. The nucleus is usually oval shaped and moderately pleomorphic, occasionally with prominent nucleoli. Additionally, multinucleated and bizarre cells could be observed, as well as many mitoses up to 12/10 HPF. In addition, plenty of vascular neoplastic emboli (Fig. 2) in the tunica albuginea and in sections taken from the testicular areas surrounding tissue were observed. After thorough sampling, very few atrophic testicular tubules were observed at the tumor periphery. Immunohistochemical analysis showed intense diffuse expression of *CD56 (NCAM1)*, patchy intense positivity for synaptophysin (Fig. 3) and negative results for *PLAP (PLAA)*, *CD30 (TNFRSF8)*, *CK8/18 (KRT18P18)*, *CK7 (KRT7)*, *CK20 (KRT20)*, desmin, myogenin, and chromogranin. S100 protein revealed the presence of spindle cells at the periphery of the neoplastic nests, similar to the sustentacular cell pattern of a paraganglioma.

**Figure 4:**
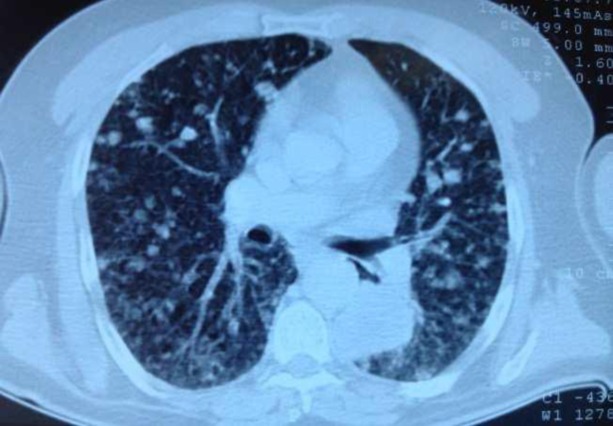
Chest CT slide highlighting distant lung metastasis (left paraspinally).

After the diagnosis of the paraganglioma, the patient was readmitted to the hospital for further investigations. Plasma and serum levels of epinephrine and norepinephrine were in the normal range and the tumor markers negative. The 24 h urine collection showed normal levels of vanillylmandelic acid. The ultrasound scan of the adrenal glands was unremarkable. The patient also underwent computed tomography of the brain, cervix, thorax, abdomen, and pelvis. The scan showed the following features:

- Multiple nodular lesions in both lungs with the larger one of diameter 4.1 cm (Fig. 4) and located on the left lower lobe paraspinally.
- Nodular lesion left subdiaphragmatically=2 cm (possible lymph node).
- No inguinal lymph nodes.
- No fractures.
- No sign of primary lesion in the adrenal glands or any other sites.

Owing to the absence of any lesion in the adrenal glands or any other sites toward the neural crest, primary paraganglioma was diagnosed in the right testicle. The patient was then referred to the oncologists for further treatment, however he refused any further treatment or intervention and passed away three months later due to myocardial infarction.

## Discussion

Based on our literature search, this is the first reported paraganglioma sited exclusively in the testis and not in the spermatic cord where the vast majority of the paratesticular tumors are found, giving birth in this way to a new subcategory of testicular tumors (3) (4). Moreover, although these tumors have been reported to be benign in 90% of the cases, in our case the lesion was aggressive with malignant characteristics, high mitotic activity, and multiple distant metastases in the lungs. From the most common symptoms of the extra-adrenal chromaffin cell tumors such as hypertension, palpitation, sweating, headache, tachycardia, and anxiety, only the former and the latter were present at the time of the surgery.

The authors of previous case reports have supported the finding that the small size of the paragangliomas in their cases along with the consequent low ability to secrete high quantities of catecholamines was responsible for the absence of significant hemodynamic symptoms.

In this case, although the size of the tumor was much bigger than all the other reported paratesticular paragangliomas, the absence of important hemodynamic variances along with the normal levels of the catecholamines in the serum and the urine is remarkable. Maybe the extra-adrenal chromaffin cell tumors located especially in the paratesticular areas as well as those in the testis lack the ability to secrete high amounts of catecholamines regardless of their size. However, the distant metastasis is a finding that can be related to the large size of the tumor in our case and indicates that early detection and excision of these tumors can be critical for the proper management and the survival of these patients.

## Patient consent

The patient's consent could not be obtained before his death.

## Author contribution statement

M C Makris, K C Koumarelas, and A S Mitrousias wrote the paper, M C Makris and E Yettimis designed, supervised, and edited the paper, G G Psathas and A Mantzioros performed literature search and data extraction, and S P Sakellariou and P Ntailiani performed immunohistological examination of the specimen.
